# Mitochondrial fat oxidation is essential for lipid-induced inflammation in skeletal muscle in mice

**DOI:** 10.1038/srep37941

**Published:** 2016-11-28

**Authors:** Jaycob D. Warfel, Estrellita M. Bermudez, Tamra M. Mendoza, Sujoy Ghosh, Jingying Zhang, Carrie M. Elks, Randall Mynatt, Bolormaa Vandanmagsar

**Affiliations:** 1 Gene Nutrient Interactions Laboratory, Pennington Biomedical Research Center, Louisiana State University System, Baton Rouge, Louisiana, USA; 2 Computational Biology Laboratory, Pennington Biomedical Research Center, Louisiana State University System, Baton Rouge, Louisiana, USA; 3 Centre for Computational Biology and Program in Cardiovascular and Metabolic Disorders, Duke-NUS Graduate Medical School, Singapore, Singapore; 4 Transgenic Core Facility, Pennington Biomedical Research Center, Louisiana State University System, Baton Rouge, Louisiana, USA; 5 Matrix Biology Laboratory, Pennington Biomedical Research Center, Louisiana State University System, Baton Rouge, Louisiana, USA.

## Abstract

Inflammation, lipotoxicity and mitochondrial dysfunction have been implicated in the pathogenesis of obesity-induced insulin resistance and type 2 diabetes. However, how these factors are intertwined in the development of obesity/insulin resistance remains unclear. Here, we examine the role of mitochondrial fat oxidation on lipid-induced inflammation in skeletal muscle. We used skeletal muscle-specific *Cpt1b* knockout mouse model where the inhibition of mitochondrial fatty acid oxidation results in accumulation of lipid metabolites in muscle and elevated circulating free fatty acids. Gene expression of pro-inflammatory cytokines, chemokines, and cytokine- and members of TLR-signalling pathways were decreased in *Cpt1b*^m−/−^ muscle. Inflammatory signalling pathways were not activated when evaluated by multiplex and immunoblot analysis. In addition, the inflammatory response to fatty acids was reduced in primary muscle cells derived from *Cpt1b*^m−/−^ mice. Gene expression of *Cd11c*, the M1 macrophage marker, was decreased; while *Cd206*, the M2 macrophage marker, was increased in skeletal muscle of *Cpt1b*^m−/−^ mice. Finally, expression of pro-inflammatory markers was decreased in white adipose tissue of *Cpt1b*^m−/−^ mice. We show that the inflammatory response elicited by elevated intracellular lipids in skeletal muscle is repressed in *Cpt1b*^m−/−^ mice, strongly supporting the hypothesis that mitochondrial processing of fatty acids is essential for the lipid-induction of inflammation in muscle.

Insulin resistance is tightly associated with obesity and is an essential part of type 2 diabetes and characterized by decreased glucose uptake in insulin-responsive organs^1^. In obesity, elevated level of free fatty acids in circulation is associated with insulin resistance^2^,^3^. Also accumulation of intracellular lipids such as ceramides and diacylglycerol (DAG) are linked to impaired insulin signalling in skeletal muscle^4^,^5^,^6^,^7^,^8^. In addition, mitochondrial dysfunction with reduced or incomplete mitochondrial fatty acid oxidation (FAO) is associated with diminished insulin signalling^9^,^10^,^11^,^12^,^13^,^14^.

Obesity is also characterized by the development of chronic inflammation in multiple tissues that contributes to insulin resistance^15^,^16^. Dietary factors like saturated fatty acids have been proposed as triggers of metabolic inflammation. The consequences include production of pro-inflammatory cytokines and the recruitment of pro-inflammatory macrophages and lymphocytes to metabolic tissues^17^,^18^,^19^,^20^. Inflammatory cytokines activate several kinases such as IKKβ and JNK which interfere with insulin signalling in myocytes, hepatocytes, and adipocytes^15^,^21^,^22^. However, how lipotoxicity and inflammation are intertwined in the pathogenesis of insulin resistance in obesity continues to remain elusive.

Carnitine palmitoyltransferase-1 (CPT1) is an enzyme located on the outer mitochondrial membrane that transports long-chain fatty acids into mitochondria for β-oxidation, thus controlling the rate of mitochondrial fatty acid oxidation (FAO). We recently described knockout mouse model with skeletal muscle specific *Cpt1b* depletion (*Cpt1b*^m−/−^) that represent a model of FAO impairment and lipid accumulation in skeletal muscle^23^. The physiological characterization of *Cpt1b*^m−/−^ mice has revealed many factors associated with obesity and insulin resistance, such as elevated levels of circulating free fatty acids and intramyocellular lipid (IMCL)^23^. Though *Cpt1b*^m−/−^ mice clearly demonstrate diminished mitochondrial fat oxidation capacity and elevated lipid levels, they do not develop insulin resistance and have attenuated adiposity relative to control mice^23^.

We have shown that the mTORC-Akt signalling pathway contributes to the increased expression of FGF21 in skeletal muscle of *Cpt1b*^m−/−^ mice, resulting in enhanced glucose utilization and insulin sensitivity^24^. However, extensive investigation as to the inflammatory status of *Cpt1b*^m−/−^ mice has not yet been reported. Since these mice maintain biological markers associated with obesity and insulin resistance without developing obesity and diabetic phenotype, the study of inflammation in *Cpt1b*^m−/−^ mice, one of the major mechanisms in the pathogenesis of obesity-associated metabolic diseases, provides an opportunity to enhance the understanding of the relationship between obesity-induced inflammation and insulin resistance.

In the present study, we address whether lipotoxicity and reduced mitochondrial FAO in skeletal muscle contribute to obesity-associated inflammation using *Cpt1b*^m−/−^ mice. We demonstrate that *Cpt1b*^m−/−^ mice fed moderate fat diet do not manifest inflammation at the systemic level. Moreover, pro-inflammatory markers, inflammatory sensing, signalling and response are reduced in skeletal muscle, which may contribute to the maintenance of insulin sensitivity of *Cpt1b*^m−/−^ mice.

## Results

### Inhibition of mitochondrial fat oxidation in skeletal muscle prevents a local inflammatory response in *Cpt1b* ^m−/−^ mice

**Figure 1:**
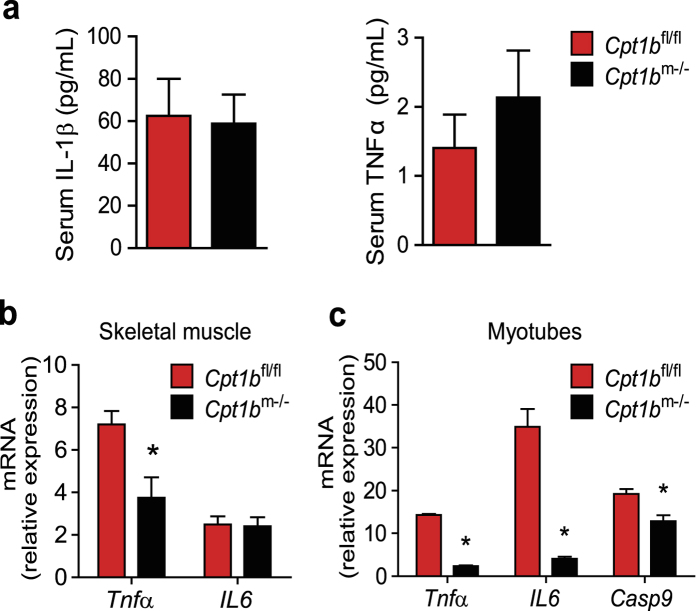
Inhibition of mitochondrial fat oxidation in skeletal muscle does not induce inflammatory response in *Cpt1b*^m−/−^ mice. (**a**) Serum levels of IL1β and TNFα; n = 6–8/group. (**b**) Relative gene expression of *Tnfα* and *IL6* in gastrocnemius muscle measured by qPCR; n = 8/group. (**c**) FA-induced inflammatory gene expression in mouse primary muscle cells; n = 3/group. Data are means ± SEM; *p < 0.05 significance for *Cpt1b*^m−/−^ mice vs control *Cpt1b*^fl/fl^ mice.

*Cpt1b*^m−/−^ mice have elevated plasma lipids, and accumulation of both intramyocellular lipid (IMCL) and lipotoxic species such as DAG and ceramides, but they have lower fasting insulin and glucose, improved glucose tolerance, and no impairment of insulin signalling in skeletal muscle^23^. We examined whether lipid overload in *Cpt1b*^m−/−^ mice induced inflammation in skeletal muscle and at the systemic level. Interestingly, serum levels of interleukin 1, IL1β and tumor necrosis factor alpha, TNFα did not differ between chow diet (CHD)-fed *Cpt1b*^m−/−^ and CHD-fed control *Cpt1b*^fl/fl^ mice (Fig. 1a). More interestingly, gene expression of pro-inflammatory cytokine *Tnfα* and chemokine C-C motif ligand 24, *Ccl24* was decreased in skeletal muscle tissue of *Cpt1b*^m−/−^ mice compared to control mice without changes in expression of interleukin 6, *IL6* (Fig. 1b and Table 1). Global analysis of gene expression and Gene Set Enrichment Analysis (GSEA) in gastrocnemius muscle revealed that expression of pro-inflammatory cytokines and chemokines which are typically increased in obese state^22^,^25^,^26^,^27^,^28^ such as chemokine (C-C motif) ligands 2, 6, 7, 9, 11 (*Ccl2 (Mcp1*), *Ccl6, Ccl7 (Mcp3*), *Ccl9, Ccl11*) and chemokine (C-X-C motif) ligands 1, 9, 12 (*Cxcl1, Cxcl9, Cxcl12*), and their receptors, *IL6ra, Ccr1, Ccr2, Ccr3*, and *Cxcr4* was not changed in muscle tissue of *Cpt1b*^m−/−^ mice compared to *Cpt1b*^fl/fl^ mice (Supplementary Table S1). Also the same was true for IL1β receptors *IL1r1* and *IL1r2*, and *TNFα* receptors *Tnfr1* and *Tnfr2* (Supplementary Table S1).

In line with this, fatty acid (FA)-induced expression of *Tnfα, IL6*, and *Casp9* was significantly decreased in *Cpt1b*^m−/−^ primary myotubes compared to *Cpt1b*^fl/fl^ myotubes, indicating that the inflammatory response to fatty acids is reduced in muscle cells from CHD-fed *Cpt1b*^m−/−^ mice (Fig. 1c). Notably, expression of *Tnfα* and *Casp9* was also significantly decreased in skeletal muscle tissue of high fat-diet (HFD) and low fat-diet (LFD) fed *Cpt1b*^m−/−^ mice compared to HFD and LFD fed *Cpt1b*^fl/fl^ mice, respectively (Supplementary Fig. S1a,b). However, expression of *IL6* was significantly decreased only in gastrocnemius muscle of HFD fed *Cpt1b*^m−/−^ mice, whereas it was not different in muscle tissue of LFD fed *Cpt1b*^m−/−^ and *Cpt1b*^fl/fl^ mice (Supplementary Fig. S1a,b). Taken together, these data suggest that inflammatory status is improved in skeletal muscle in *Cpt1b*^m−/−^ mice despite the presence of excess lipids at the systemic and tissue levels.

### TLR-signalling is downregulated in skeletal muscle from *Cpt1b* ^m−/−^ mice

**Figure 2:**
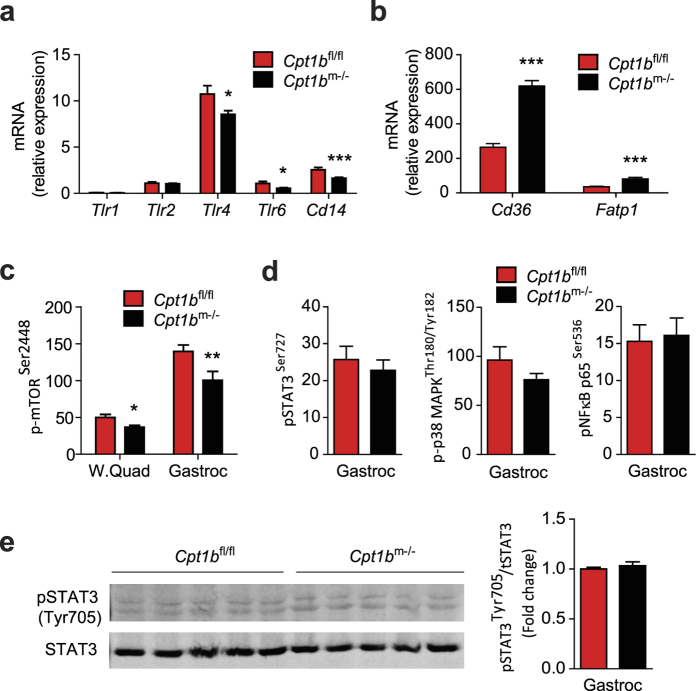
Inflammatory signaling pathways are not activated in muscle of *Cpt1b*^m−/−^ mice. (**a,b**) Relative gene expression of TLR-members and *Cd14* (**a**), and fatty acid transport proteins *Cd36, Fatp1* (**b**) in gastrocnemius muscle of *Cpt1b*^fl/fl^ and *Cpt1b*^m−/−^ mice measured by qPCR; n = 8/group. (**c,d**) Activity of mTOR (**c**), and STAT3, p38 MAPK, and NFkB (**d**) pathways in gastrocnemius muscle of *Cpt1b*^fl/fl^ and *Cpt1b*^m−/−^ mice evaluated by multiplex protein assay; n = 5–11/group. (**e**) Activation of STAT3 pathway as examined by phosphorylation at Tyr 705 in gastrocnemius muscle of *Cpt1b*^fl/fl^ and *Cpt1b*^m−/−^ mice evaluated by western blot analysis (left). Image J software was used for densitometry quantification of the immunoblots (right); n = 5/group. Data are means ± SEM; *p < 0.05 and **p < 0.01 and ***p < 0.005 significances for *Cpt1b*^m−/−^ vs control *Cpt1b*^fl/fl^ mice.

Toll-like receptors (TLRs) are involved in bridging the immune response to metabolic disturbances as a nutrient sensor and as a part of inflammatory signalling^29^,^30^,^31^. Increased amounts of FA directly induce Toll-like receptors (TLRs)^25^,^32^. LPS, a metabolic endotoxin is also increased in obese, insulin resistant mice, and exacerbates inflammation via the TLR4-signalling^33^. Next, we examined whether excess circulating FA stimulate TLR-signalling in muscle from *Cpt1b*^m−/−^ mice. The expression of *Tlr4, Tlr6*, and *Cd14*, the receptor for LPS-binding protein was significantly decreased in skeletal muscle from CHD fed *Cpt1b*^m−/−^ mice compared to CHD fed control mice (Fig. 2a and Table 1). Interestingly, gene expression of FA-transport proteins, *Cd36* and *Fatp1* was increased in *Cpt1b*^m−/−^ muscle, suggesting that FA overload on TLRs and FA uptake in *Cpt1b*^m−/−^ muscle are increased even in CHD condition compared to control mice (Fig. 2b). More interestingly, expression of *Tlr4* and *Cd14* was also significantly lower in muscle tissue from *Cpt1b*^m−/−^ mice compared to *Cpt1b*^fl/fl^ mice without changes in expression of *Tlr6* when mice were challenged with HFD (Supplementary Fig. S1a). However, expression of *Tlr1* and *Tlr*2 in muscle was not different between CHD fed *Cpt1b*^m−/−^ and *Cpt1b*^fl/fl^ mice (Fig. 2a). Also, expression of *Tlr4, Tlr6*, and *Cd14* was not changed in muscle of LFD fed *Cpt1b*^m−/−^ mice compared to LFD fed control mice (Supplementary Fig. S1b). Together, these results suggest that the activity of TLR-signalling pathway is decreased in skeletal muscle of *Cpt1b*^m−/−^ mice despite increased levels of FA in circulation and consequently an excessive load of FA in muscle.

### Inflammatory signalling pathways are not activated in *Cpt1b* deficient muscle

Next we examined activation of inflammatory pathways in skeletal muscle of *Cpt1b*^m−/−^ mice using Multiplex Mapmate assays. The mTORC1 pathway (the mammalian target of rapamycin, mTOR and its downstream target, p70 ribosomal S6 kinase or p70S6K) is involved in nutrient sensing and inflammatory signalling^34^. Notably, the activity of mTORC1 signalling was decreased in white quad and gastrocnemius muscle in *Cpt1b*^m−/−^ mice compared to control mice (Fig. 2c). In the obese state, increased levels of fatty acids and cytokines in circulation induce activation of inflammatory signalling pathways such as STAT3 (signal transducer and activator of transcription 3), p38 MAPK (p38 mitogen-activated protein kinases) and NFkB (nuclear factor kappa-light-chain-enhancer of activated B cells) in metabolic tissue^35^,^36^. However, the activity of the inflammatory signalling pathways was not different in gastrocnemius muscle of *Cpt1b*^m−/−^ mice compared to *Cpt1b*^fl/fl^ mice (Fig. 2d).

In addition to phosphorylation at Ser 727, STAT3 is activated through phosphorylation at Tyr 705 in response to various cytokines including IFNs (interferons), IL5 and IL6 (interleukins 5 and 6)^37^,^38^,^39^. However, immunoblot analysis revealed that basal activation of STAT3 through Tyr 705 – phosphorylation in gastrocnemius muscle was similar between *Cpt1b*^fl/fl^ and *Cpt1b*^m−/−^ mice (Fig. 2e).

In line with this, global gene expression analysis and GSEA analyses revealed that expression of intracellular signal transducers involved in TNF-mediated activation of NFkB, MAPK and JNK (c-Jun N-terminal kinases) pathways such as *Cd27* and *Traf1* (TNF receptor associated factor 1), and other members of TNFR-signalling pathways such as *Casp3* and *Casp9* were significantly decreased in skeletal muscle of *Cpt1b*^m−/−^ mice compared to *Cpt1b*^fl/fl^ mice (Table 1). Tab1 (TGF-beta activated kinase 1/MAP3K7 binding protein 1), Tbkbp1 (TBK1 binding protein 1), Tradd (TNF receptor type 1-associated DEATH domain protein) and Traf2 (TNF receptor associated factor 2) are members of TNFα/NFkB signal transduction network^40^. Expression of *Tab1* and *Tbkbp1* was significantly reduced in *Cpt1b*^m−/−^ muscle, whereas expression of *Tradd* and *Traf2* in skeletal muscle was not different between *Cpt1b*^m−/−^ and *Cpt1b*^fl/fl^ mice (Table 1 and Supplementary Table S1).

Leukotriene B4 (LTB4) enhances macrophage chemotaxis and stimulates inflammatory pathways, and is increased in liver, muscle, and adipose tissue in HFD-fed obese mice, and directly promotes insulin resistance^41^. Inhibition of the LTB4 receptor, Ltb4r1 leads to an anti-inflammatory phenotype and insulin sensitizing effects^41^. Interestingly, expression of *Ltb4r1* was significantly decreased in gastrocnemius muscle from *Cpt1b*^m−/−^ mice compared to control mice (Table 1). However, expression of *IL1rap*, which is involved in IL1-signaling, and *IL6st*, an IL6 – signal transducer was not different in muscle tissue between *Cpt1b*^m−/−^ and *Cpt1b*^fl/fl^ mice (Supplementary Table S1).

**Figure 3:**
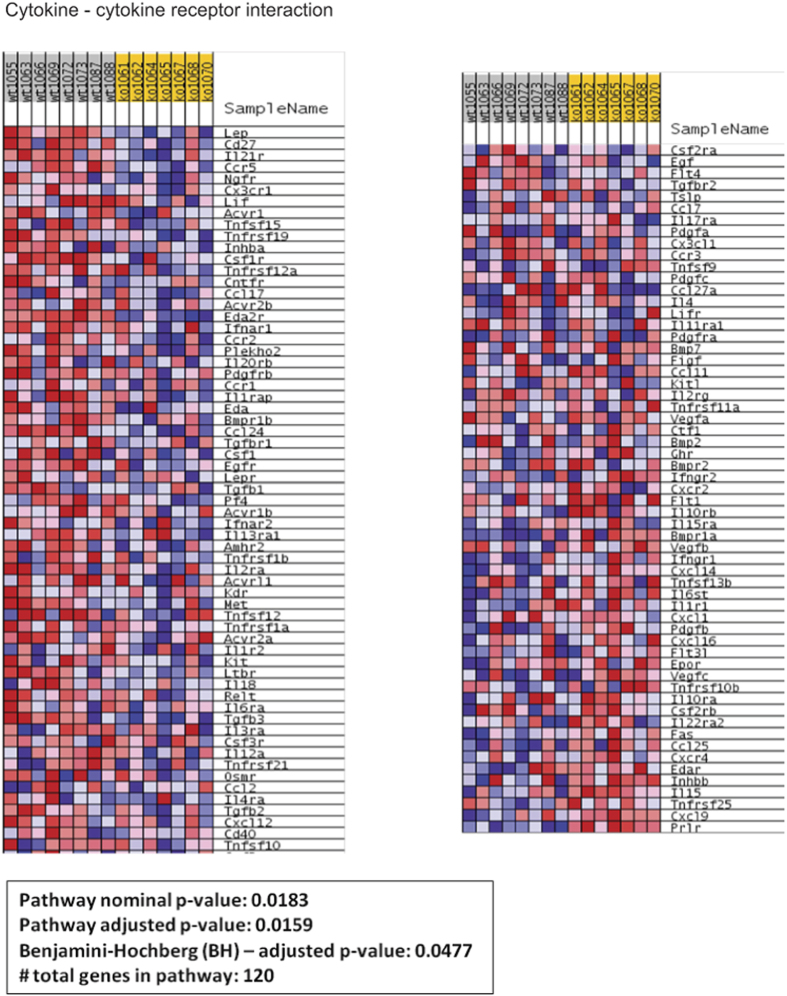
Cytokine-Cytokine receptor interaction pathway related gene expression pattern determined by Ingenuity Pathway Analysis (IPA) and Gene Set Enrichment Analysis (GSEA) in gastrocnemius muscle from *Cpt1b*^fl/fl^ (wt) and *Cpt1b*^m−/−^ (ko) mice, n = 7–8/group.

**Figure 4:**
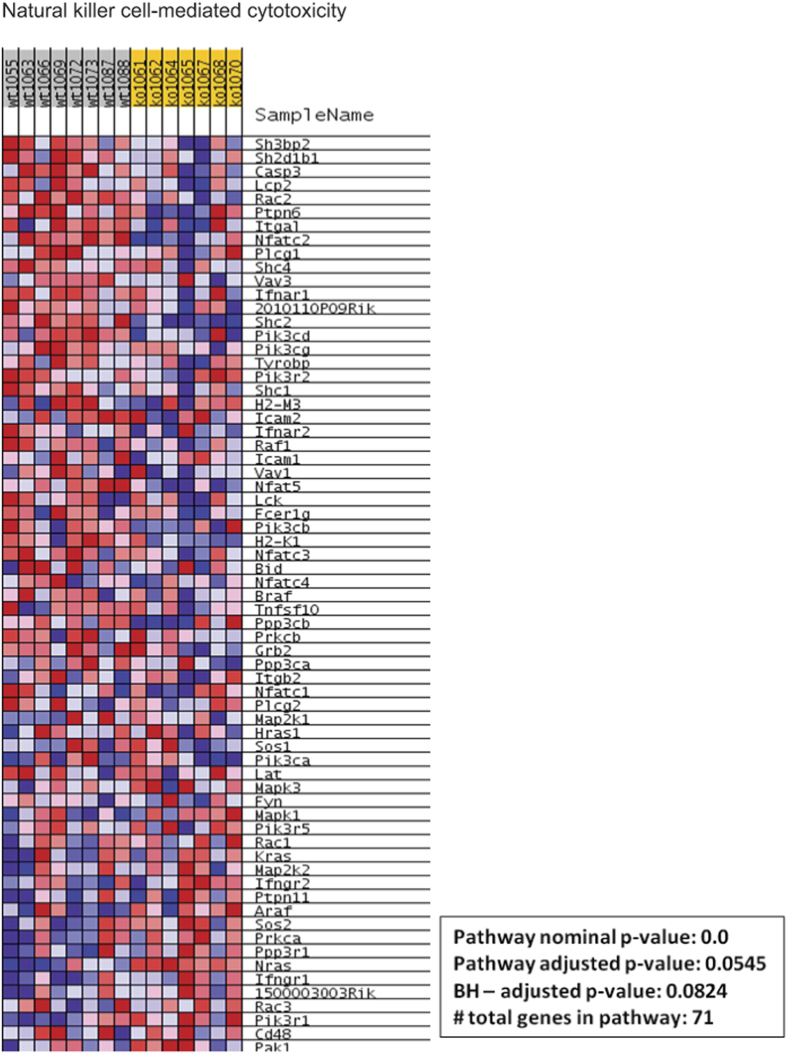
Natural killer cell-mediated cytotoxicity related gene expression pattern determined by Ingenuity Pathway Analysis (IPA) and Gene Set Enrichment Analysis (GSEA) in gastrocnemius muscle from *Cpt1b*^fl/fl^ (wt) and *Cpt1b*^m−/−^ (ko) mice, n = 7–8/group.

**Figure 5:**
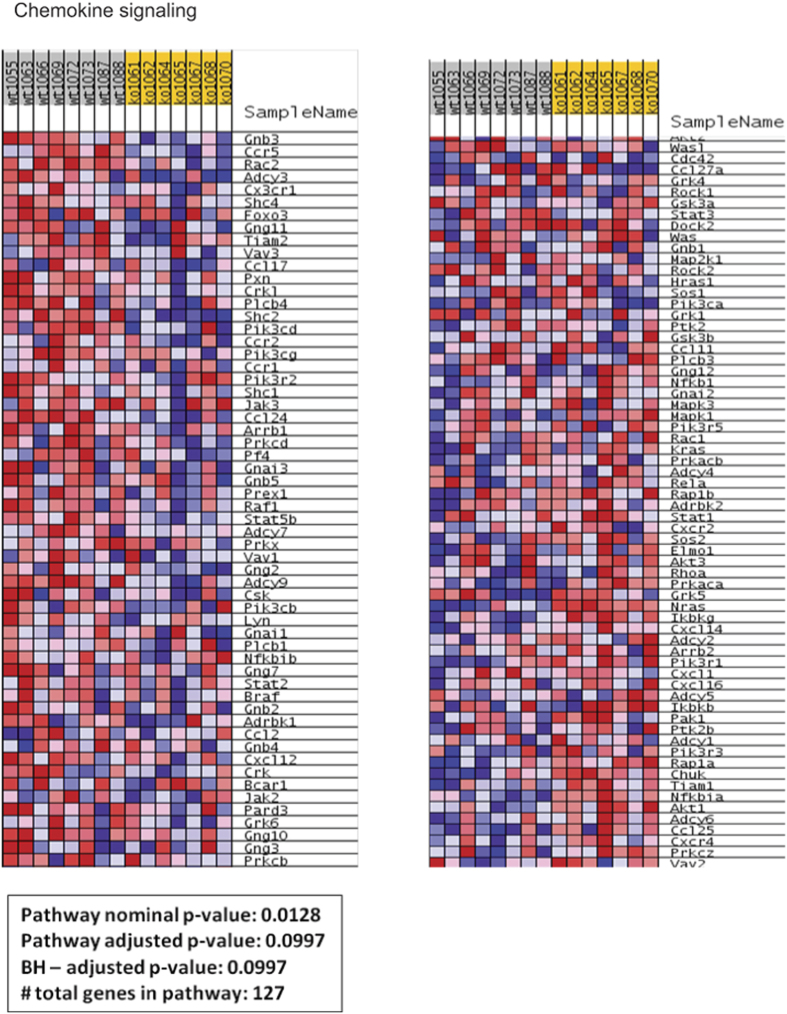
Chemokine signaling related gene expression pattern determined by Ingenuity Pathway Analysis (IPA) and Gene Set Enrichment Analysis (GSEA) in gastrocnemius muscle from *Cpt1b*^fl/fl^ (wt) and *Cpt1b*^m−/−^ (ko) mice, n = 7–8/group.

In addition to the evaluation of these selected genes, analysis of inflammation-associated pathways based on GSEA results obtained in gastrocnemius muscle from *Cpt1b*^m−/−^ and control *Cpt1b*^fl/fl^ mice revealed that cytokine-cytokine receptor interaction pathways were significantly downregulated in muscle of *Cpt1b*^m−/−^ mice (Fig. 3 and Supplementary Figs S2 and S3). Though the GSEA results and enrichment plot suggested that the natural killer (NK) cell-mediated cytotoxicity pathway is downregulated in muscle of *Cpt1b*^m−/−^ mice, the pathway adjusted p-value was at the borderline of significance (Fig. 4 and Supplementary Figs S4 and S5). Also, the chemokine signalling pathway was not different between *Cpt1b*^m−/−^ and *Cpt1b*^fl/fl^ mice (Fig. 5 and Supplementary Fig. S6).

Taken together, these data indicate that cytokine-induced inflammatory signalling pathways are not activated in skeletal muscle of *Cpt1b*^m−/−^ mice. Consistent with the reduction in expression of components which are involved in TNFα-mediated intracellular signal transduction, *TNFα* expression is decreased in *Cpt1b*^m−/−^ muscle. However, expression of members involved in IL1and IL6 signaling pathways is unaffected in *Cpt1b*^m−/−^ muscle and, as a result, expression of *IL1β* and *IL6* is not changed in *Cpt1b*^m−/−^ muscle.

### *Cpt1b* ablation shifts immune cell function toward anti-inflammatory in skeletal muscle

**Figure 6:**
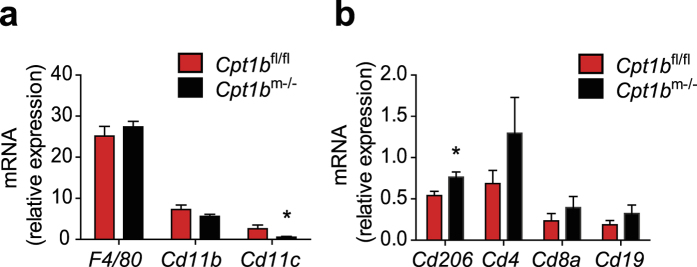
Immune cell markers are not elevated in skeletal muscle of *Cpt1b*^m−/−^ mice. (**a,b**) Relative gene expression of *F4/80, Cd11b, Cd11c* (**a**), and *Cd206, Cd4, Cd8a, Cd19* (**b**) in gasrocnemius muscle of *Cpt1b*^fl/fl^ and *Cpt1b*^m−/−^ mice measured by qPCR; n = 8/group. Data are means ± SEM; *p < 0.05 and **p < 0.01 and ***p < 0.005 significances for *Cpt1b*^m−/−^ vs control *Cpt1b*^fl/fl^ mice.

Another major characteristic of chronic inflammation in obesity is increased infiltration of pro-inflammatory immune cells in metabolic tissue when no source of infection or trauma is present^42^. To gain insight into whether immune cells in skeletal muscle of *Cpt1b*^m−/−^ mice contribute to reduction in inflammation in *Cpt1b* deficient muscle tissue, we evaluated immune cell populations by gene expression of cell markers in muscle. CD11c^+^ cells are classical pro-inflammatory M1 macrophages that are activated by FA^43^,^44^, whereas CD206^+^ cells are known as anti-inflammatory oriented M2 macrophages^43^,^45^,^46^. Notably, expression of *Cd11c* was significantly decreased (Fig. 6a), while expression of *Cd206* was significantly increased in skeletal muscle of *Cpt1b*^m−/−^ mice compared to *Cpt1b*^fl/fl^ mice when mice were fed CHD (Fig. 6b). In addition, expression of *Lyz2 (LyzM*), a marker of mature myeloid cells such as monocytes, macrophages and neutrophils^47^ was significantly decreased in *Cpt1b*^m−/−^ muscle (Table 1). However, expression of other pro-inflammatory macrophage markers *F4/80* and *Cd11b*, and T cell markers *Cd4* and *Cd8a*, and B cell marker *Cd19* was unchanged in *Cpt1b*^m−/−^ muscle compared to control *Cpt1b*^fl/fl^ muscle (Fig. 6).

In HFD fed mice, expression of pro-inflammatory macrophage markers, *F4/80* and *Cd11c* was significantly decreased in *Cpt1b*^m−/−^ muscle compared to control *Cpt1b*^fl/fl^ muscle, without change in expression of *Cd206* (Supplementary Fig. S1a). However, in LFD fed mice, none of the immune cell markers *F4/80, Cd11c*, and *Cd206* were changed in *Cpt1b*^m−/−^ muscle tissue compared to control mice (Supplementary Fig. S1b). Taken together, in contrast to obese mice, infiltration of immune cells is not enhanced in *Cpt1b*^m−/−^ muscle despite excess amounts of metabolic stimuli such as FA. Instead, a shift from M1 to M2 macrophages could favour reduced inflammation in *Cpt1b* deficient muscle.

### Changes in pro-inflammatory gene expression is not associated with fiber type in skeletal muscle of *Cpt1b* ^m−/−^ mice

Skeletal muscle consists of complex mixture of fibers, and different isoforms of myosin heavy chain (Myh) determine type 1 or 2 fibers^48^,^49^. It has been demonstrated that resting healthy human muscles express cytokines in a fiber type specific manner^50^. Pro-inflammatory cytokines TNFα and IL18 were exclusively expressed by type 2 fibers, whereas expression of IL6 was largely observed in type 1 fibers. We examined whether *Cpt1b*-deficiency affects fiber type composition and therefore plays a role in expression of inflammatory markers in muscle. We determined the distribution of the four major fiber types: 1, 2a, 2x, and 2b in skeletal muscle using expression of genes *Myh7* (MHC I), *Myh2* (MHC IIa), *Myh1* (MHC IIx), and *Myh4* (MHC IIb), respectively (Supplementary Fig. S7). Expression of *Myh7* was significantly increased and *Myh4* was significantly decreased in gastrocnemius muscle of *Cpt1b*^m−/−^ mice compared to *Cpt1b*^fl/fl^ mice without changes in *Myh2* and *Myh1* expression, suggesting a possible fiber-type switch from type 2b to type 1 in *Cpt1b* deficient muscle.

Interestingly, a statistical correlation analysis revealed that expression of *Tnfα* was positively and significantly associated with expression of type 2a, 2x and 2b fiber genes in skeletal muscle of control *Cpt1b*^fl/fl^ mice (Supplementary Fig. S8). Notably, *Tnfα* expression was not associated with expression of any fiber-type genes in gastrocnemius muscle of *Cpt1b*^m−/−^ mice. However, expression of *IL6* was not associated with expression of any fiber-type specific genes in either, control *Cpt1b*^fl/fl^ or *Cpt1b*^m−/−^ mice (Supplementary Fig. S9). In addition to pro-inflammatory cytokines, correlation analysis revealed no association between expression of *Tlr4* and immune cell markers *F4/80, Cd11c* and *Cd206* with fiber-type specific genes in muscle of control *Cpt1b*^fl/fl^ mice and *Cpt1b* deficient mice (data not shown).

Taken together, *Cpt1b*^m−/−^ muscle has more type 1 fibers and less type 2b fibers, however, it appears that fiber type distribution or fiber-switching does not affect expression of inflammatory markers or immune cell infiltration in skeletal muscle of *Cpt1b*^m−/−^ mice.

### Inflammatory status is improved in adipose tissue of *Cpt1b* ^m−/−^ mice

**Figure 7:**
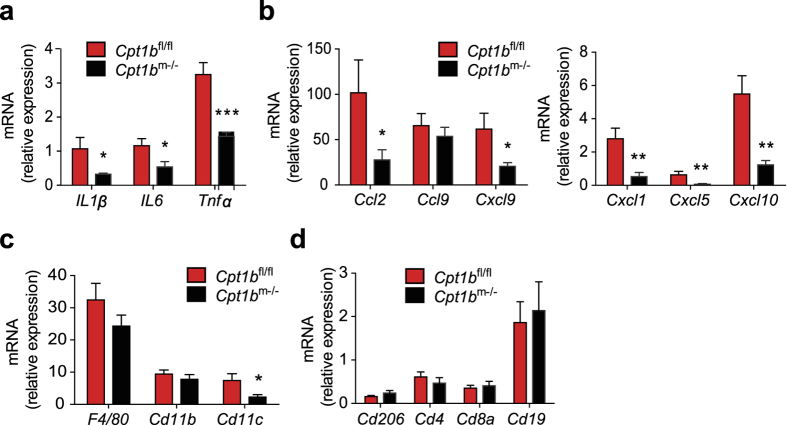
Inflammatory status is improved in adipose tissue of *Cpt1b*^m−/−^ mice. (**a,b**) Relative gene expression of *IL1β, IL6, Tnfα* (**a**) and chemokines (**b**) in epididymal white adipose tissue (eWAT) of *Cpt1b*^fl/fl^ and *Cpt1b*^m−/−^ mice measured by qPCR; n = 8/group. Data are means ± SEM; *p < 0.05 and **p < 0.01 and ***p < 0.005 significances for *Cpt1b*^m−/−^ vs control *Cpt1b*^fl/fl^ mice.

Adipose tissue is considered to be a main source of inflammation in obesity with increased production of pro-inflammatory cytokines and chemokines and infiltration of pro-inflammatory immune cells^19^,^42^,^43^,^51^,^52^. We detected significantly decreased expression of cytokines *IL1β, IL6* and *Tnfα*, and chemokines *Ccl2, Cxcl9, Cxcl1, Cxcl5* and *Cxcl10* in epididymal white adipose tissue (eWAT) of *Cpt1b*^m−/−^ mice compared to control *Cpt1b*^fl/fl^ mice, whereas expression of *Ccl9* was not different in these mice (Fig. 7a,b).

The expression of macrophage markers *F4/80, Cd11b*, and *Cd206*, T cell markers *Cd4* and *Cd8a*, and B lymphocyte marker *Cd19* was not different in adipose tissue of *Cpt1b*^m−/−^ mice compared to *Cpt1b*^fl/fl^ mice (Fig. 7c,d). Notably, as a result of reduced production of chemokines including Ccl2 and Cxcl10, which recruit monocytes to the site of inflammation, adipose tissue expression of M1 macrophage marker *Cd11c* was decreased in eWAT from *Cpt1b*^m−/−^ mice compared to control *Cpt1b*^fl/fl^ mice (Fig. 7c). Taken together, decrease in expression of inflammatory markers such as pro-inflammatory cytokines and chemokines and infiltration of pro-inflammatory macrophages in adipose tissue of *Cpt1b*^m−/−^ mice indicates that inflammatory status is improved in adipose tissue despite the elevated levels of lipids in circulation.

## Discussion

Obesity-induced chronic inflammation is triggered by metabolic signals such as excess nutrients, and involves inflammatory responses originated within metabolic cells such as adipocytes, hepatocytes or myocytes, resulting in damage to metabolic homeostasis with insulin resistance^16^,^17^,^18^,^36^,^42^,^53^,^54^,^55^. In agreement with these studies, we have previously found activated NFkB pathway and increased expression of pro-inflammatory genes in insulin resistant myotubes derived from diabetic-obese subjects, but not in insulin sensitive myotubes derived from non-diabetic-lean subjects^56^. Thus, our data suggest that excess fat is sufficient to activate inflammatory signalling pathways in skeletal muscle resulting in elevated chemoattractant chemokines that in turn increase infiltration of pro-inflammatory immune cells in muscle.

*Cpt1b*^m−/−^ mice recapitulate a model of increased ectopic fat accumulation both in serum and in skeletal muscle. We have previously shown that when *Cpt1b* is knocked out in skeletal muscle, mice have diminished mitochondrial oxidative capacity of dietary fat^23^. Surprisingly, this decrease in muscle mitochondrial function results in a lean, insulin sensitive phenotype characterized by decreased serum insulin and body weight due to reduced fat mass. *Cpt1b*^m−/−^ mice on a CHD (25% calorie from fat) display behavioural differences from controls, including decreased food intake and decreased activity^23^.

Despite a decrease in dietary intake, *Cpt1b*^m−/−^ mice still maintain higher levels than controls of systemic and tissue lipids, which have classically been shown to be triggers of inflammatory response^25^,^32^. Given that *Cpt1b*^m−/−^ mice have these metabolic stressors even on CHD (25% calorie from fat), lipid-induced inflammation would be expected in this model. However, we demonstrate here that *Cpt1b*^m−/−^ mice do not manifest inflammation in skeletal muscle or at the systemic level and do not possess a strong inflammatory response to the presence of elevated fatty acids. Sensing and signalling mechanisms which stand at the intersection of metabolic and inflammatory pathways such as TLRs, mTOR, JNK, MAPK, and NFkB that are activated in obesity-associated inflammation^57^,^58^,^59^, were not induced in *Cpt1b*^m−/−^ muscle. Moreover, the inflammatory response to excess lipids is decreased in *Cpt1b*-deficient muscle cells possibly leading to favourable changes in chemokine production that in turn results in a switch to anti-inflammatory function of immune cells in skeletal muscle of *Cpt1b*^m−/−^ mice. Also, the fact that inflammatory response and recruitment of inflammatory immune cells in skeletal muscle of *Cpt1b*^m−/−^ mice remained significantly lower compared to *Cpt1b*^fl/fl^ mice even when mice were fed HFD for a prolonged time suggest that the depletion of *Cpt1b* itself promotes a reduction of inflammatory markers independent of the diet. Furthermore, similar levels in expression of inflammatory markers such as TLR-signalling members and immune cell markers in muscle of LFD fed *Cpt1b*^m−/−^ mice and LFD fed control mice suggest that depletion of CPT1b indeed keeps inflammatory status in muscle as low as at the LFD level (10% calorie from fat) even in mice fed CHD (25% calorie from fat) or HFD (45% calorie from fat).

Though the presence of elevated systemic and IMCL would tend to predict higher levels of inflammation, some of our previously reported results fall in line with the improved inflammatory status of *Cpt1b*^m−/−^ mice. One key contributor to the activation of inflammatory signalling pathways and the inflammatory response in obese state is the metabolic stress of organelles such as mitochondria and endoplasmic reticulum (ER)^42^. However, previously we reported that mitochondrial and ER stress levels were not different in muscle between *Cpt1b*^m−/−^ and control mice^24^.

It has been shown that skeletal muscle produces cytokines dependent upon contraction^60^,^61^,^62^,^63^. Though a decreased expression of inflammatory markers was not associated with fiber type switching in *Cpt1b*^m−/−^ muscle, a reduced activity of *Cpt1b*^m−/−^ mice could contribute to a reduction of contraction-induced cytokine response.

The beneficial effects of *Cpt1b* deficiency in muscle on inflammation are observed beyond skeletal muscle. Adipose tissue inflammation largely contributes to obesity-induced pro-inflammatory state and insulin resistance^19^,^22^,^25^,^43^,^52^. We found that inflammation in adipose tissue with increased pro-inflammatory cytokines and chemokines, and infiltrated immune cells in obesity is absent in *Cpt1b*^m−/−^ mice. We have reported that inhibition of mitochondrial fat oxidation in muscle induces FGF21 specifically in skeletal muscle of *Cpt1b*^m−/−^ mice^24^. FGF21 is also secreted into circulation as a myokine in *Cpt1b*^m−/−^ mice and promotes browning of inguinal white adipose tissue (iWAT), but not eWAT in *Cpt1b*^m−/−^ mice^24^. Recent reports have shown that FGF21 decreased expression of IL6 and TNFα in adipose tissue of obese rats and suppressed inflammation in mouse models with FA-induced or diabetic renal dysfunction^64^,^65^. Thus muscle derived FGF21 in *Cpt1b*^m−/−^ mice could have anti-inflammatory action locally in the presence of intracellular toxic lipids, and on adipose or possibly other tissues by circulation contributing to the improvement in inflammatory status at the face of constantly elevated systemic lipids.

Emerging evidence suggests the existence of a close relationship between metabolic and inflammatory systems^5^,^15^,^22^,^66^,^67^. Our data show a coordinated decrease in mitochondrial processing of fatty acids and expression of inflammatory marker genes in skeletal muscle of *Cpt1b*^m−/−^ mice. On the other hand, we previously reported that pathways that are involved in amino acid, citric acid (TCA cycle), pyruvate and fatty acid metabolism, oxidative phosphorylation, and peroxisome function are upregulated in *Cpt1b*^m−/−^ muscle^23^. Skeletal muscle uses the oxidation of lipids as a fuel during fasting periods and switches to the oxidation of carbohydrate during fed periods. Proper switching between different fuels is impaired in insulin resistant skeletal muscle^68^,^69^. Notably, *Cpt1b*^m−/−^ mice successfully switch to usage of carbohydrates and amino acids, and use peroxisomal FAO to rescue decreased mitochondrial FAO in skeletal muscle^23^. Accordingly, one explanation is that decreased inflammatory activity might be compensatory to increased metabolic function in *Cpt1b*^m−/−^ muscle. In line with this, we have reported increased expression of genes that are associated with mitochondrial branched-chain amino acid (BCAA) cycle such as *Bckdha* and *Bcat2*, and citric acid or tricarboxylic acid (TCA) cycle such as *Cs* and *Pdha1* with concomitant increase in leucine and pyruvate oxidation in *Cpt1b*^m−/−^ muscle^23^. Previous studies have indicated that higher levels of TNFα are associated with decreased expression of BCAA/TCA cycle genes^70^. The lower levels of TNFα expression reported in our study are therefore consistent with the observed enhanced expression of BCAA and TCA cycle genes in *Cpt1b*^m−/−^ mice.

In summary, our data suggest that elevated lipids within models of obesity and insulin resistance are not alone sufficient to induce chronic inflammation. Instead, metabolic status and homeostasis between metabolic systems and inflammatory mechanisms that prevents lipid-induced stress upon various cellular organelles such as the ER and mitochondria is important in the development of chronic inflammation in obesity. Specifically, our results presented here suggest that inhibition of CPT1 in skeletal muscle is protective against muscle and systemic inflammation despite the presence of excess lipid stressors possibly due to metabolic compensatory mechanisms developed to rescue impaired mitochondrial fat oxidation in skeletal muscle.

## Methods

### Animals

Animal studies were conducted at Pennington Biomedical Research Center’s AALAC-approved facility using skeletal muscle specific *Cpt1b*^m−/−^ knockout mice where deletion of *Cpt1b* gene is driven by *Mlc1f* promoter^23^. *Cpt1b*^m−/−^ and control *Cpt1b*^fl/fl^ mice have C57Bl6 background^23^,^71^. All experiments were in compliance with the NIH Guide for the Care and Use of Laboratory Animals, and approved by the Institutional Animal Care and Use Committee at Pennington Biomedical Research Center. All mice utilized in the experiments were 5–6 month old males. Age matched *Cpt1b*^m−/−^ mice and control *Cpt1b*^fl/fl^ mice were fed a breeder’s chow diet consisting of 25% calories from fat (Purina Rodent Chow no. 5015, Purina Mills, St. Louis, MO, USA). Also *Cpt1b*^m−/−^ and *Cpt1b*^fl/fl^ mice were fed a high fat-diet consisting of 45% calories from fat (D12451, Research Diets Inc, New Brunswick, NJ, USA) starting at 8 week of age for 16 weeks prior to the experiments, whereas mice from low fat-diet group were fed a chow diet consisting of 10% calories from fat (D12450, Research Diets Inc, New Brunswick, NJ, USA) starting at weaning age until they were utilized for experiments.

### Mouse primary muscle cell culture

Cultures were established from mixed hindlimb muscle of 1 month old *Cpt1b*^m−/−^ and *Cpt1b*^fl/fl^ littermates^24^. Collagenase digestion was used to isolate satellite cells (0.5% collagenase B, 1.2 U/ml Dispase II (Roche) in Ham’s F-10 media (Thermo Scientific)), and enrichment in non-collagen coated flasks before initiation of culture and between passages used to reduce fibroblast content. Cells were maintained in collagen-coated flasks in Rat Skeletal Muscle Cell Growth Medium (Cell Applications, Inc, San Diego, CA, USA) and myoblasts at passage 3 were used for experiments. Briefly, the myoblasts at passage 2 were subcultured onto 24 well plates and grown to 80–90% confluence. Cells were then differentiated into fused multinucleated myotubes in Ham’s F-10 media with 2% horse serum for 5–7 days. Myotubes were treated with either essentially fatty-acid free BSA or BSA-conjugated palmitate:oleate (1:1 ratio, total 0.5 mM) plus 2.5 mM carnitine. Treatments were performed in serum-free MEM alpha media with nucleosides (Gibco, Waltham, MA, USA) for 24 h and cells were harvested for gene expression analysis. At least three independent cultures were performed for each gene expression analysis by qRT-PCR and multiple wells (at least three replicates) were used per treatment.

### Gene expression analysis

Total RNA from mouse tissue was isolated using RNeasy Mini Kit (Qiagen, Valencia, CA, USA) and total RNA from mouse primary myotubes was isolated using RNeasy Micro Kit (Qiagen, Valencia, CA, USA). All samples were DNase digested to remove potential genomic DNA contamination. cDNA was synthesized with the iScript cDNA synthesis kit and used for qRT-PCR with the SYBR Green system (Bio-Rad, Hercules, CA, USA). qRT-PCR was conducted using ∆∆C_T_ assays. Mouse cyclophilin B was used for normalization of gene expression. Primer details are provided in Supplementary Table 2.

### Serial Analysis of Gene Expression (SAGE)

1–2 ug of total RNA extracted from mouse gastrocnemius muscle was used to perform the SAGE analysis as previously described^72^. Briefly, gene expression profiling was performed by expression tag sequencing (SAGE) on an AB SOLiD 5500XL next-generation sequencing instrument using reagent kits from the manufacturer (Applied Biosystems, Foster City, CA). Sequence reads were aligned to mouse RefSeq transcripts (version mm9) as the reference, utilizing the program SOLiDSAGE (Applied Biosystems). Only uniquely mapped sequence reads were counted to generate the expression count level for each respective RefSeq gene.

### Global gene expression analysis and Gene Set Enrichment Analysis

Pathway enrichment was conducted via the gene set enrichment analysis procedure (GSEA) based on ranks^73^. GSEA was performed by first weighting (ranking) the muscle gene expression data for the wild-type and Cpt1b−/− groups via a signal-to-noise ratio (SNR) metric, and then employing a weighted Kolmogorov-Smirnov test to determine if the gene SNRs deviate significantly from a uniform distribution in a priori defined gene-sets (pathways). In our studies, these gene-sets were obtained from the Kyoto Encyclopedia of Genes and Genomes or KEGG^74^ via the Molecular Signatures Database repository (MSigDb, http://software.broadinstitute.org/gsea/msigdb/). Statistical significance of the observed enrichment was ascertained by permutation testing over size-matched gene-sets. Significant gene-sets were selected by control of the false discovery rate, FDR at 25%^75^. The per-sample expression profiles of genes contributing to core enrichment of the significant pathways were visualized via row-normalized blue-red heatmaps with blue representing lower, and red representing higher gene expression levels.

### Multiplex analysis

Serum collections in mice were performed by submandibular bleed using BD microtainer SST (Becton, Dickinson and Company, Franklin Lakes, NJ, USA). Analytes (beads) for IL-1β and TNFα (Bio-Rad, Hercules, CA, USA) were prepared according to the manufacturer instructions and Bio-Plex Mouse Cytokine Assay kit (Bio-Rad, Hercules, CA, USA) was used for serum multiplex assay.

Harvested muscle tissues from mice were snap frozen in liquid nitrogen. Muscle tissue was then powdered in liquid nitrogen and used for protein lysate preparation in Cell Signaling Lysis Buffer (Millipore, Billerica, MA, USA). Total protein lysates from mouse tissue powder were prepared according to the manufacturer’s instructions and used for Multiplex Mapmate signaling assays. The following mouse Mapmates were used for cytokine signalling assay: p-JNK (Thr183/Tyr185), p-p38 MAPK (Thr180/Tyr182), p-mTOR (Ser2448) (Millipore); and p-STAT3 (Ser727), pNFκB p65 (Ser536), p-P70S6K (Thr421/Ser424) (Bio-Rad). Milliplex Map Cell signaling buffer and Detection kits (Millipore) were used in all multiplex signalling assays and Map mates were prepared and combined according to the manufacturer instructions. 10 μg of mouse tissue protein lysate was used in each assay and assays were run in duplicate. Mean fluorescence intensity (MFI) of phospho-proteins was measured on a Luminex 200 Analyzer (Millipore) and analyzed using Milliplex Analyst Software (Millipore).

### Western blot analysis

Gastrocnemius homogenates were prepared in a non-denaturing buffer (2% Triton X-100, 300 mM NaCl, 20 mM Tris (pH 7.4), 2 mM EDTA, 2 mM EGTA, and 1% NP-40) containing the following phosphatase and protease inhibitors: 1 mM PMSF, 10 uM leupeptin, 38 uM aprotinin, 1 mM phenanthrolin, 1 uM pepstatin, 1 mM sodium fluoride, and 200 uM sodium vanadate. Total protein concentrations were measured using a bicinchoninic acid assay (ThermoFisher Scientific). Western blot analysis (200 ug protein/well) was performed using standard procedures followed by Odyssey IF detection. The antibodies used were pSTAT3 (Tyr705) from BD Transduction Laboratories and STAT3 from Santa Cruz Biotechnology. Densitometric analysis was performed using ImageJ software (NIH).

### Statistical Analyses

GraphPad Prism 5 software, Student’s *t* test and Pearson’s correlation coefficient *r* were used for statistical analysis. All data are presented as the mean ± SEM. *p* < 0.05 was considered statistically significant.

## Additional Information

**How to cite this article**: Warfel, J. D. *et al*. Mitochondrial fat oxidation is essential for lipid-induced inflammation in skeletal muscle in mice. *Sci. Rep.* **6**, 37941; doi: 10.1038/srep37941 (2016).

**Publisher's note:** Springer Nature remains neutral with regard to jurisdictional claims in published maps and institutional affiliations.

## Supplementary Material

Supplementary Information

## Figures and Tables

**Table 1:** Changes in expression of genes related to cytokine and chemokine signalling and inflammatory phenotype in *Cpt1b* ^m−/−^ muscle.

Gene symbol	Gene name	Changes	Significance
Casp3	caspase 3	↓	0.003
Casp9	caspase 9	↓	0.015
Ccl24	chemokine (C-C motif) ligand 24	↓	<0.0001
Cd27	CD27 antigene	↓	0.005
Ltb4r1	Leukotriene B4 receptor 1	↓	0.023
Lyz2 (LyzM)	Lysozyme 2	↓	0.038
Tab1	TGF-beta activated kinase 1/MAP3K7 binding protein 1	↓	0.029
Tbkbp1	TBK1 binding protein 1	↓	0.001
Tlr6	Toll like receptor 6	↓	0.019
Traf1	TNF receptor-associated factor 1	↓	0.017

Gene expression data are obtained from Serial analysis of gene expression (SAGE) and Gene set enrichment analysis (GSEA) expression datasets in gastrocnemius muscle from *Cpt1b*^fl/fl^ and *Cpt1b*^m−/−^ mice; *n* = 8 (for *Cpt1b*^fl/fl^ mice) or *n* = 7 (for *Cpt1b*^m−/−^ mice). *p* < 0.05 was considered significant.
