# Supplementary material for: Mitochondrial fat oxidation is essential for lipid-induced inflammation in skeletal muscle in mice
Source: Sci Rep. 2016 Nov 28;6:37941. doi: 10.1038/srep37941 (PMC5124994; doi:10.1038/srep37941)
Supplement: Supplementary Information [file srep37941-s1.pdf]

Supplementary Information

Mitochondrial fat oxidation is essential for lipid-induced inflammation in skeletal muscle in mice

Jaycob D Warfel[1], Estrellita M. Bermudez[1], Tamra M. Mendoza[1], Sujoy Ghosh[4,5], Jingying Zhang[2], Carrie M. Elks[3], Randall Mynatt[1,2] and Bolormaa Vandanmagsar[1*]

[1]Gene Nutrient Interactions Laboratory, [2]Transgenic Core Facility , [3]Matrix Biology Laboratory, [4]Computuional Biology Laboratory, Pennington Biomedical Research Center, Louisiana State University System, Baton Rouge, Louisiana, USA, [5]Centre for Computational Biology and Program in Cardiovascular and Metabolic Disorders, Duke-NUS Graduate Medical School, Singapore, Singapore.

[*]Corresponding author: Bolormaa Vandanmagsar

Supplementary Table S1 Expression of genes related to cytokine and chemokine signaling and inflammatory phenotype in *Cpt1b*$^{m-/-}$ muscle

Gene symbol	Full name	*P* value
	Cytokines, chemokines	
Ccl2 (Mcp1)	chemokine (C-C motif) ligand 2	0.442
Ccl6	chemokine (C-C motif) ligand 6	0.497
Ccl7 (Mcp3)	chemokine (C-C motif) ligand 7	0.565
Ccl9	chemokine (C-C motif) ligand 9	0.271
Ccl11 (eotaxin1)	chemokine (C-C motif) ligand 11	0.954
Cxcl1 (KC)	chemokine (C-X-C motif) ligand 1	0.941
Cxcl9 (MIG)	chemokine (C-X-C motif) ligand 9	0.109
Cxcl12	chemokine (C-X-C motif) ligand 12	0.385
	Receptors, members of signal transduction	
Ccr1	C-C chemokine receptor type 1, receptor for Ccl6 and Ccl9	0.486
Ccr2	C-C chemokine receptor type 2, receptor for Ccl2	0.076
Ccr3	C-C chemokine receptor type 3, receptor for Ccl24	0.505
Cxcr4	C-X-C chemokine receptor type 4, receptor for Cxcl12	0.252
IL1r1	Interleukin-1 receptor, type 1	0.574
IL1r2	Interleukin-1 receptor, type 2	0.903
IL1rap	Interleukin-1 receptor accessory protein	0.065
IL6ra (IL-6R)	Interleukin-6 receptor, alpha	0.628
IL6st (gp130)	Interleukin-6 signal transducer	0.224
Tnfrsf1a (Tnfr1)	TNF receptor superfamily member 1A	0.226
Tnfrsf1b (Tnfr2)	TNF receptor superfamily member 1B	0.249
Tradd	TNF receptor type 1-associated death domain protein	0.057
Traf2	TNF receptor-associated factor 2	0.455

Gene expression data are obtained from Serial analysis of gene expression (SAGE) and Gene set enrichment analysis (GSEA) expression dataset in gastrocnemius muscle from *Cpt1b*$^{fl/fl}$ and *Cpt1b*$^{m-/-}$ mice; n=8 (for *Cpt1b*$^{fl/fl}$ mice) or n=7 (for *Cpt1b*$^{m-/-}$ mice). $P < 0.05$ was considered significant.

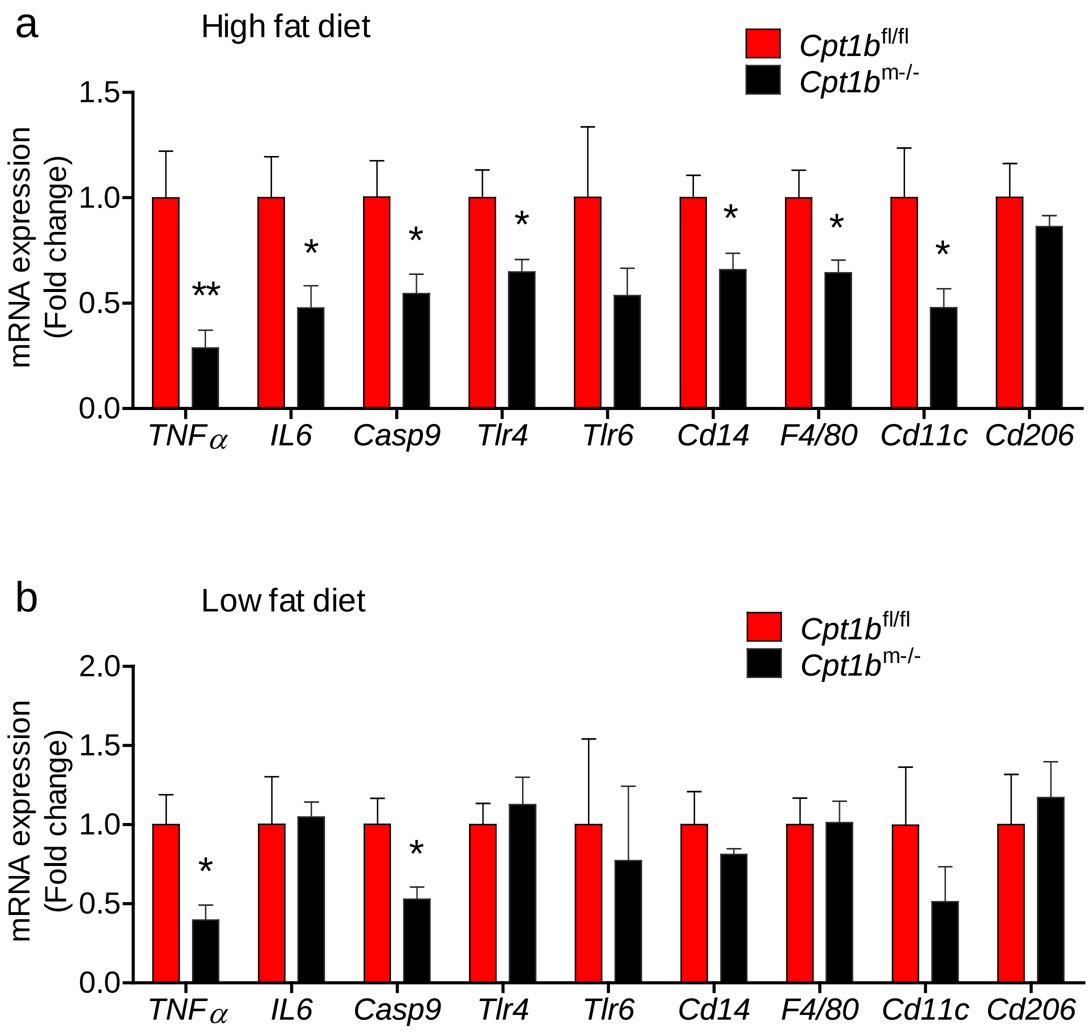

Supplementary Figure 1

Expression of pro-inflammatory markers such as cytokines *TNFα, IL6, Casp9*, and members of TLR-signaling pathways *Tlr4, Tlr6, Cd14*, and immune cells markers *F4/80, Cd11c, Cd206* in skeletal muscle of *Cpt1b*$^{fl/fl}$ and *Cpt1b*$^{m-/-}$ mice measured by qPCR. **A**. Mice were fed high fat diet for 16 weeks and then were used for experiments at 6 month of age, n = 7-8/group. **B**. Mice were fed low fat diet until they were used for experiments at 8 month of age, n = 4/group. Data are means ± SEM; *$p < 0.05$ and **$p < 0.01$ significance for *Cpt1b*$^{m-/-}$ vs control *Cpt1b*$^{fl/fl}$ mice.

Table: GSEA Results Summary

Dataset	input_gsea_data_cpt_ko1070rem_41014.input_gsea_class_cpt_ko1070rem_41014
Phenotype	input_gsea_class_cpt_ko1070rem_41014.cls
Upregulated in class	WT
GeneSet	KEGG_CYTOKINE_CYTOKINE_RECEPTOR_INTERACTION
Enrichment Score (ES)	0.36323607
Normalized Enrichment Score (NES)	1.4024113
Nominal p-value	0.018348623
FDR q-value	0.54620516
FWER p-Value	0.997

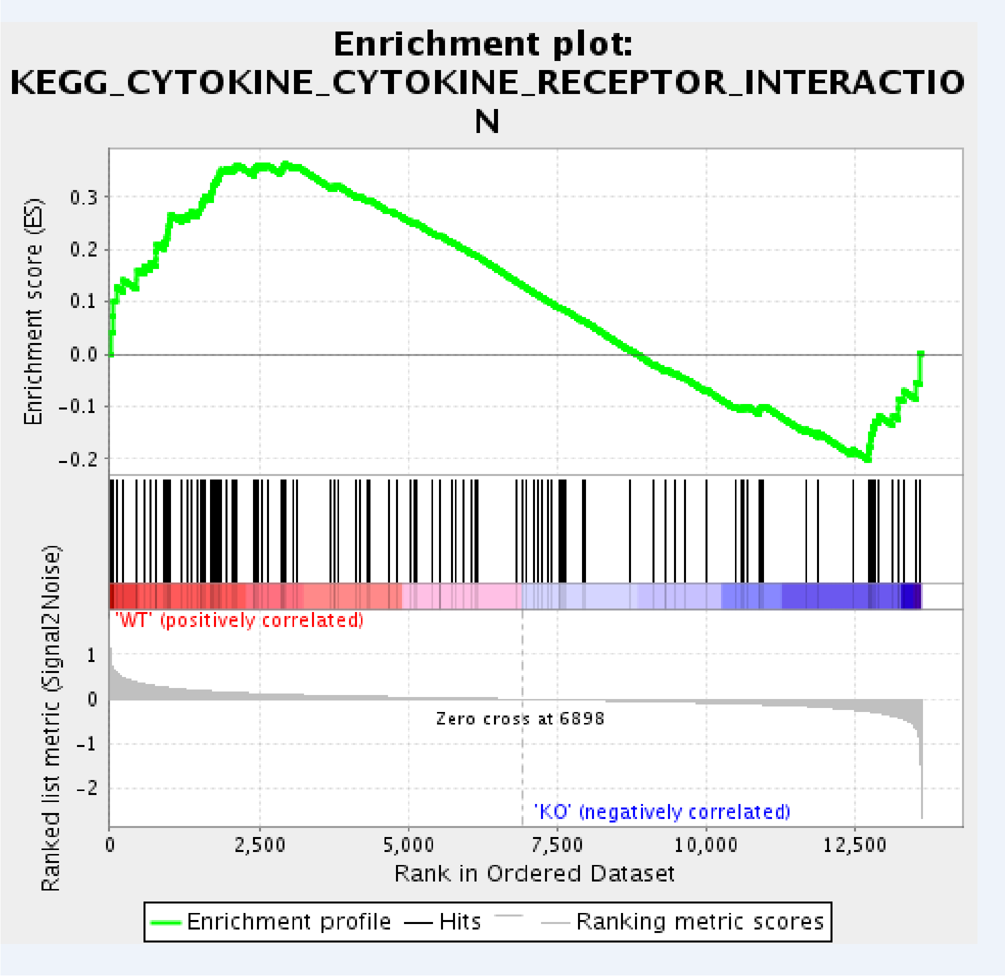

Supplementary Figure 2.

Gene Set Enrichment Analysis (GSEA) results summary and enrichment plot for KEGG Cytokine-Cytokine receptor interaction pathway. Profile of the Running Enrichment Score (ES) and positions of GeneSet Members on the rank ordered list. GSEA was performed in gastrocnemius muscle from *Cpt1b*$^{fl/fl}$ (WT) and *Cpt1b*$^{m-/-}$ (KO) mice, *n=7-8*.

Cytokine-cytokine receptor interaction

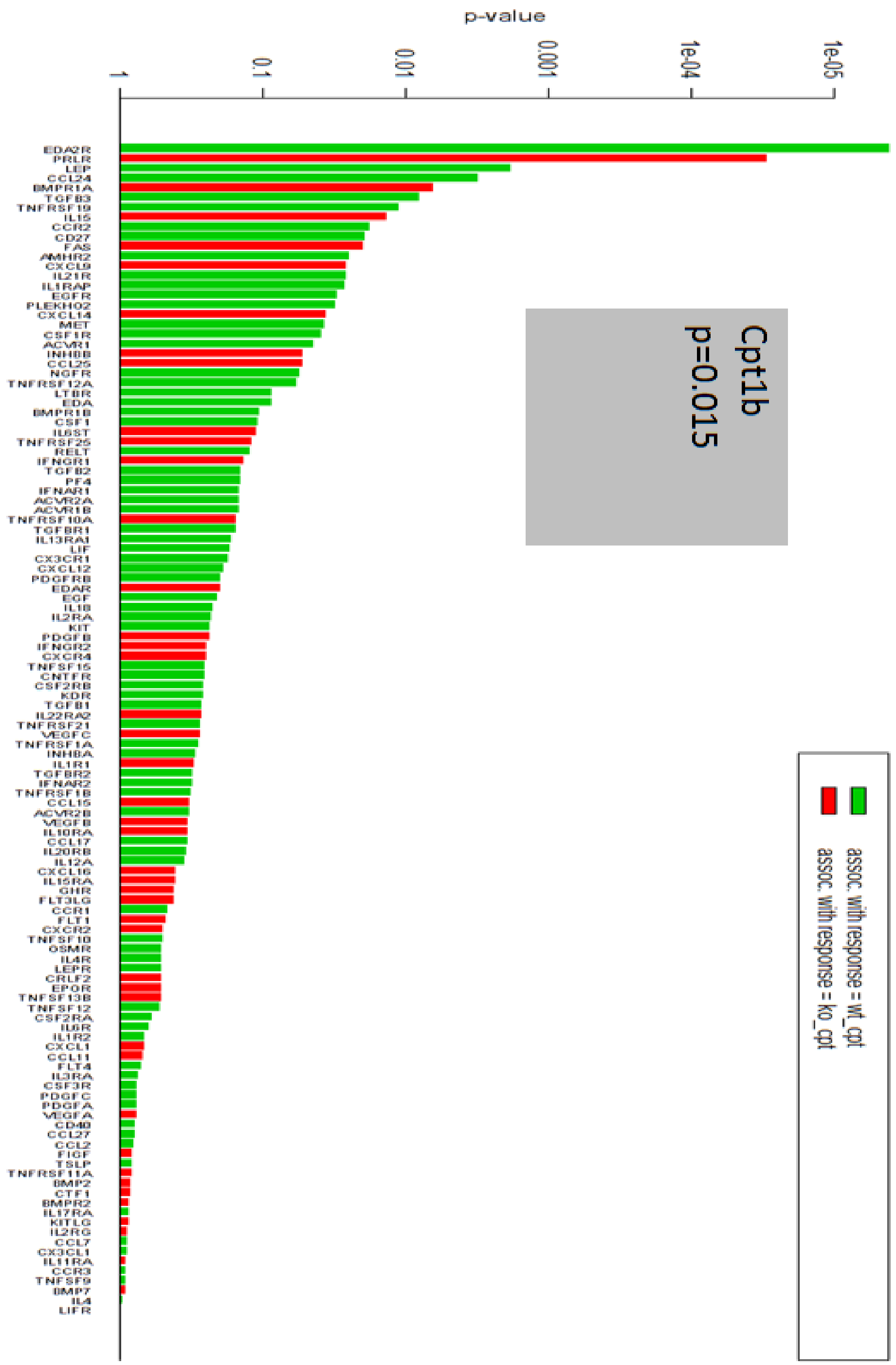

Supplementary Figure 3.

Results from globaltest (non-competitive pathway analysis method) include a pathway p-value, along with a bar plot showing the p-values of individual genes for the cytokine-cytokine receptor interaction pathway in gastrocnemius muscle from *Cpt1b*$^{fl/fl}$ (wt-labeled by green color) and *Cpt1b*$^{m-/-}$ (ko-labeled by red color) mice, *n=7-8*.

Table: GSEA Results Summary

Dataset	input_gsea_data_cpt_ko1070rem_41014.input_gsea_class_cpt_ko1070rem_41014
Phenotype	input_gsea_class_cpt_ko1070rem_41014.cls
Upregulated in class	WT
GeneSet	KEGG_NATURAL_KILLER_CELL_MEDIATED_CYTOTOXICITY
Enrichment Score (ES)	0.4557263
Normalized Enrichment Score (NES)	1.636532
Nominal p-value	0.0
FDR q-value	0.23503074
FWER p-Value	0.543

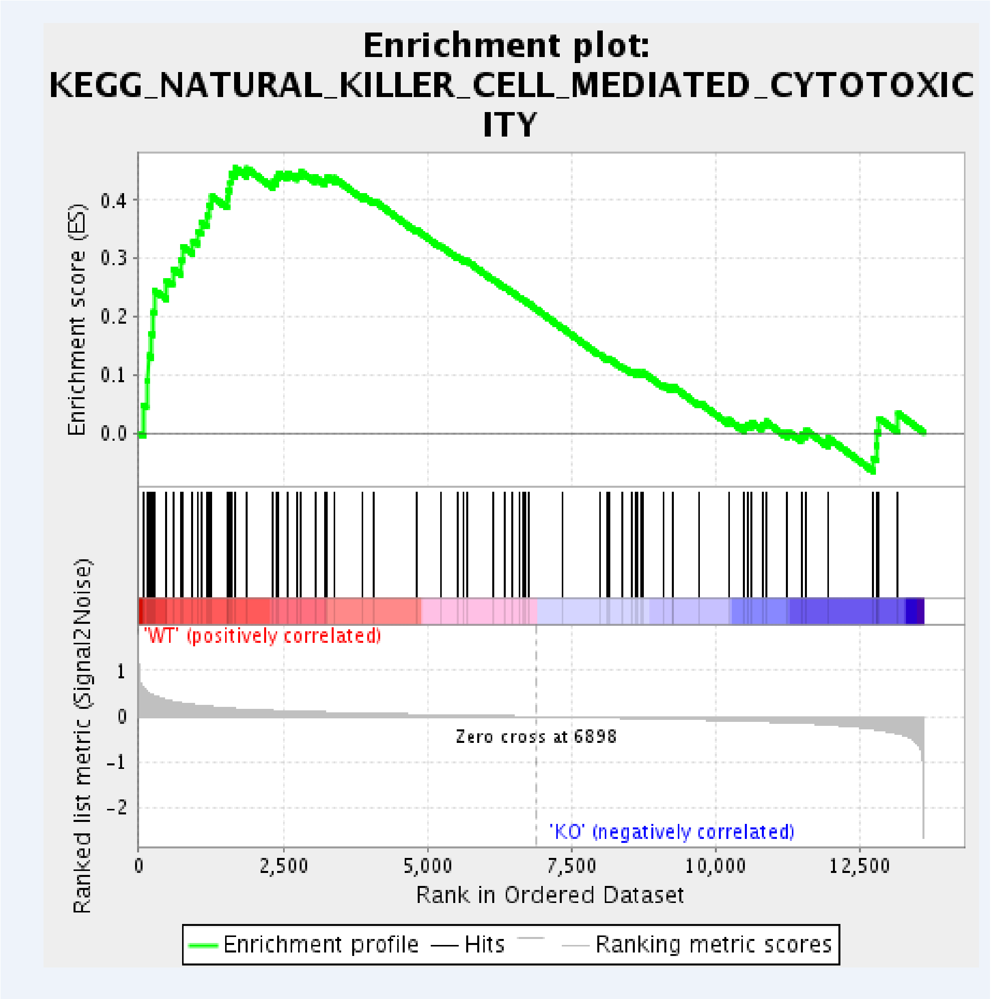

Supplementary Figure 4.

Gene Set Enrichment Analysis (GSEA) results summary and enrichment plot for KEGG Natural killer cell mediated cytotoxicity pathway. Profile of the Running ES score and positions of GeneSet Members on the rank ordered list. GSEA was performed in gastrocnemius muscle from *Cpt1b*$^{fl/fl}$ (WT) and *Cpt1b*$^{m-/-}$ (KO) mice, *n=7-8*.

Natural killer cell-mediated cytotoxicity

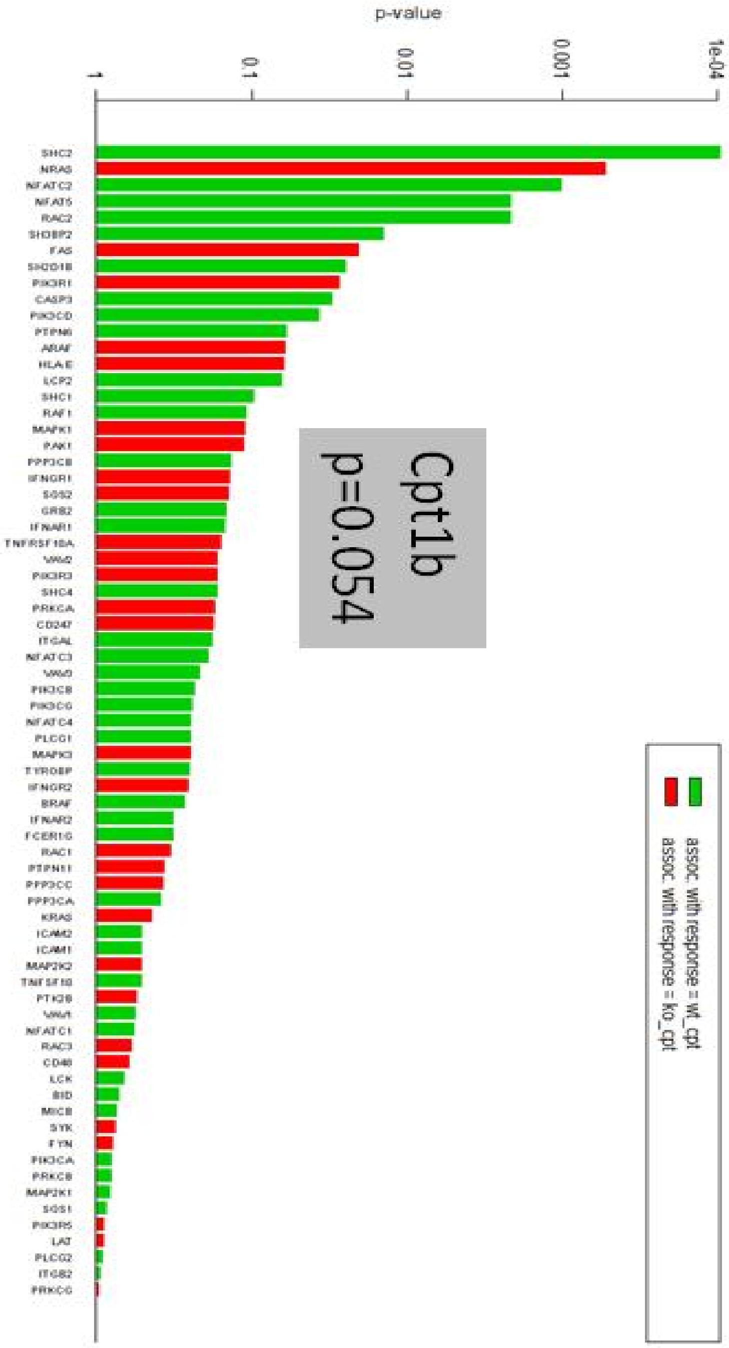

Supplementary Figure 5.

Results from globaltest (non-competitive pathway analysis method) include a pathway p-value, along with a bar plot showing the p-values of individual genes for the natural killer cell-mediated cytotoxicity pathway in gastrocnemius muscle from *Cpt1b*$^{fl/fl}$ (wt-labeled by green color) and *Cpt1b*$^{m-/-}$ (ko-labeled by red color) mice, *n=7-8*.

Table: GSEA Results Summary

Dataset	input_gsea_data_cpt_ko1070rem_41014.input_gsea_class_cpt_ko1070rem_41014
Phenotype	input_gsea_class_cpt_ko1070rem_41014.cls
Upregulated in class	WT
GeneSet	KEGG_CHEMOKINE_SIGNALING_PATHWAY
Enrichment Score (ES)	0.35354966
Normalized Enrichment Score (NES)	1.3803098
Nominal p-value	0.012820513
FDR q-value	0.57632416
FWER p-Value	0.998

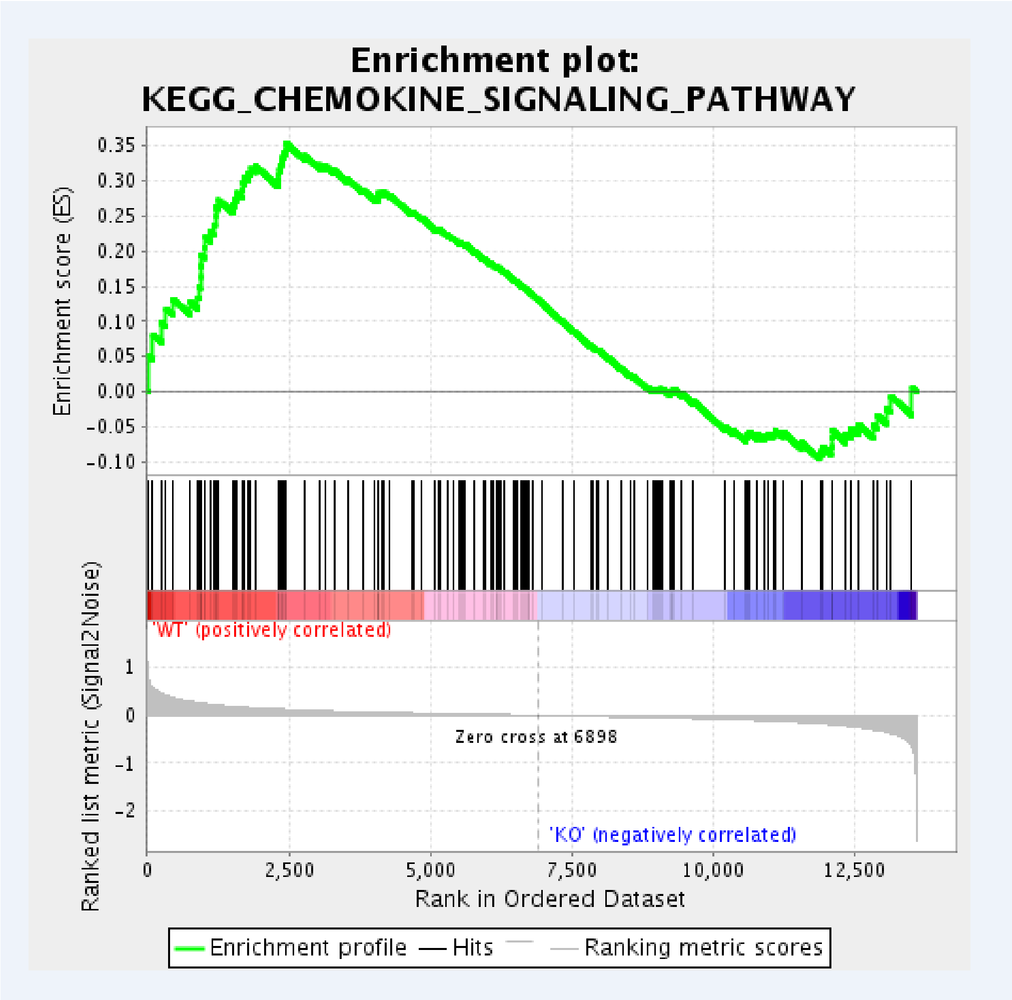

Supplementary Figure 6.

Gene Set Enrichment Analysis (GSEA) results summary and enrichment plot for KEGG Chemokine signaling pathway. Profile of the Running ES score and positions of GeneSet Members on the rank ordered list. GSEA was performed in gastrocnemius muscle from *Cpt1b*$^{fl/fl}$ (WT) and *Cpt1b*$^{m-/-}$ (KO) mice, *n=7-8*.

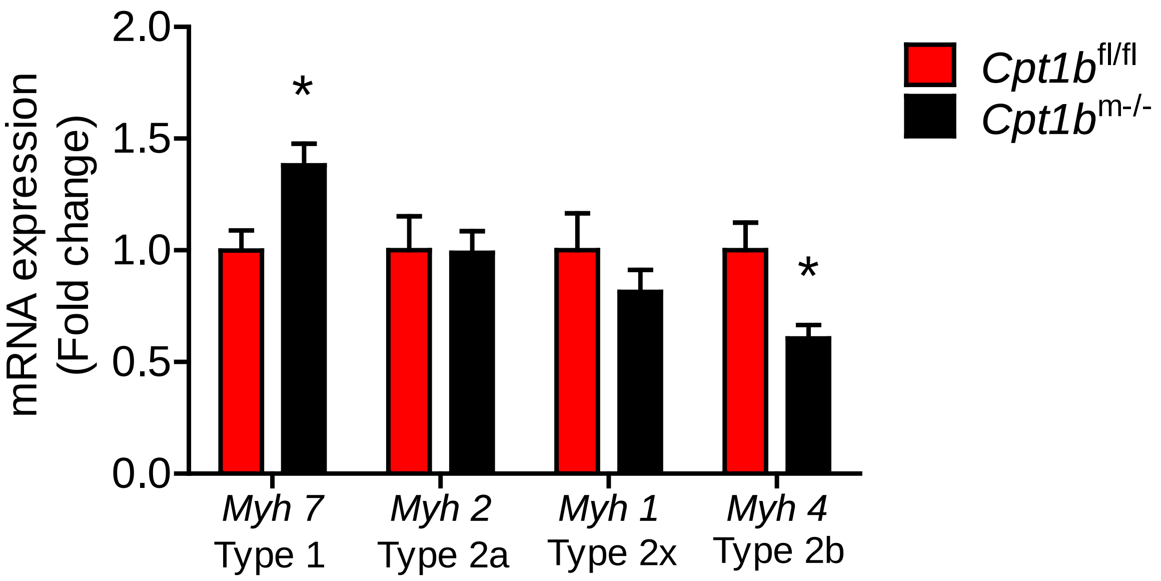

Supplementary Figure 7

Composition of fiber types in gastrocnemius muscle of *Cpt1b*$^{fl/fl}$ and *Cpt1b*$^{m-/-}$ mice measured by qPCR. Expression of genes (fold change): *Myh7*- myosin heavy chain 7 (MHC I), expressed in Type 1 fiber; *Myh2* – myosin heavy chain 2 (MHC IIa), expressed in Type 2a fiber; *Myh1* – myosin heavy chain 1 (MHC IIx), expressed in Type 2x/d fiber; *Myh4* – myosin heavy chain 4 (MHC IIb), expressed in Type 2b fiber. n = 8/group. Data are means ± SEM; *$p < 0.05$ and higher significance for *Cpt1b*$^{m-/-}$ vs control *Cpt1b*$^{fl/fl}$ mice.

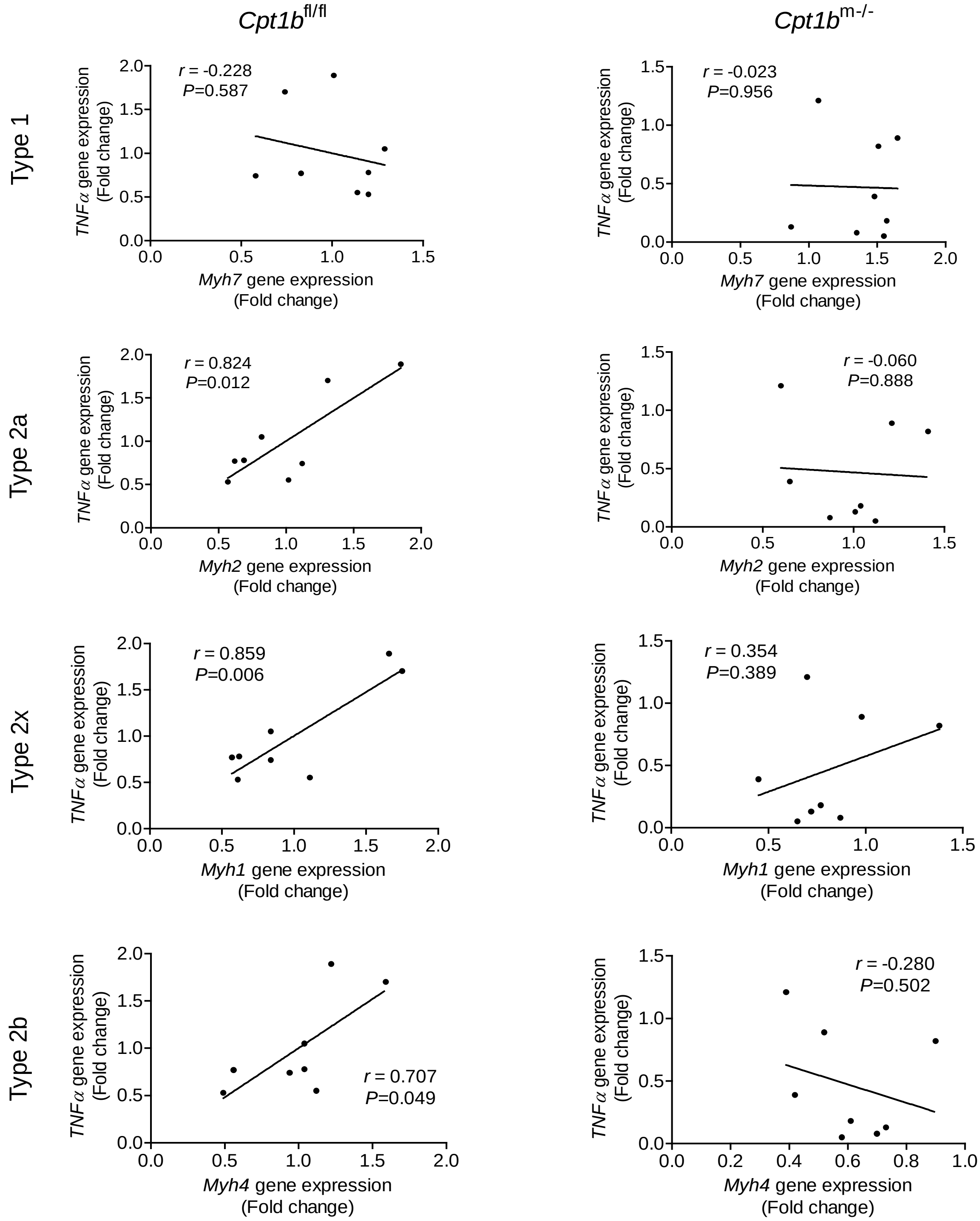

Supplementary Figure 8.

Correlation between expression of fiber type specific genes and *Tnfα* in gastrocnemius muscle of *Cpt1b*$^{fl/fl}$ and *Cpt1b*$^{m-/-}$ mice. n=8 for *Cpt1b*$^{fl/fl}$ and *Cpt1b*$^{m-/-}$ mice; *r* – Pearson's correlation coefficient; *P* – p value.

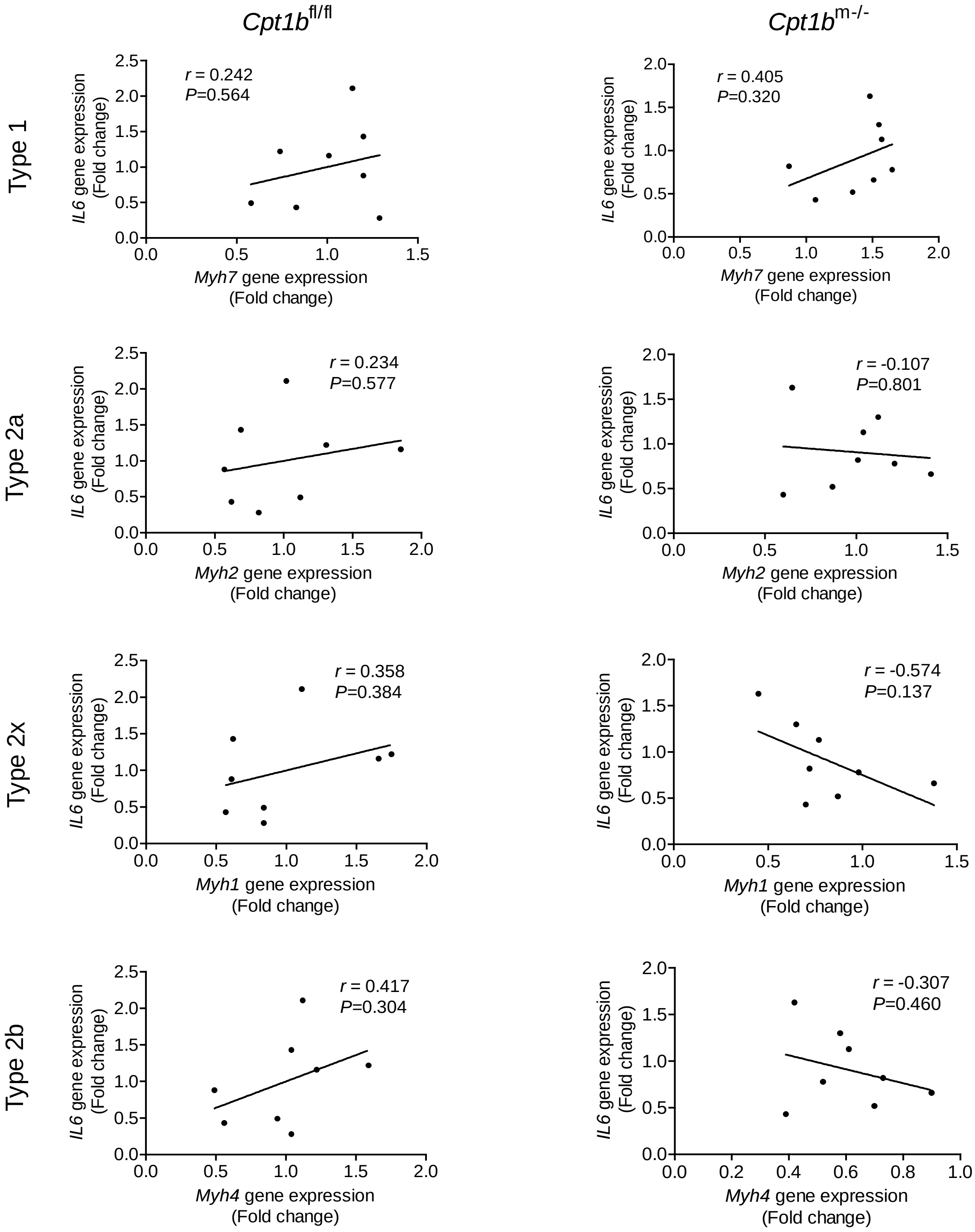

Supplementary Figure 9.

Correlation between expression of fiber type specific genes and *IL6* in gastrocnemius muscle of *Cpt1b*$^{fl/fl}$ and *Cpt1b*$^{m-/-}$ mice. n=8 for *Cpt1b*$^{fl/fl}$ and *Cpt1b*$^{m-/-}$ mice; *r* – Pearson's correlation coefficient; *P* – p value.

Supplemental Table S2: Primer Sequences for qPCR

	Gene symbol	Ref.Seq ID	Forward primer	Reverse primer	Notes
1	*Ccl2 (Mcp1)*	NM_011333	ATTGGGATCATCTTGCTGGT	CCTGCTGTTCACAGTTGCC	Mus musculus
2	*Tnf*α	NM_013693	AGGGTCTGGGCCATAGAACT	CCACCACGCTCTTCTGTCTAC	
3	*IL1*β	NM_008361	GGTCAAAGGTTTGGAAGCAG	TGTGAAATGCCACCTTTTGA	
4	*IL6*	NM_031168	ACCAGAGGAAATTTTCAATAGGC	TGATGCACTTGCAGAAAACA	
5	*Ccl9 (MIP-1g)*	NM_011338	CAGTCTGAAGGCACAGCAAG	TAGGACAGGCAGCAATCTGA	
6	*Cxcl1 (KC)*	NM_008176	TCTCCGTTACTTGGGGACAC	CCACACTCAAGAATGGTCGC	
7	*Cxcl5 (LIX)*	NM_009141	CAGAAGGAGGTCTGTCTGGA	GTGCATTCCGCTTAGCTTTC	
8	*Cxcl9 (MIG)*	NM_008599	TTTTCCTCTTGGGCATCATC	GCATCGTGCATTCCTTATCA	
9	*Cxcl10 (IP10)*	NM_021274	CGTCATTTTCTGCCTCATCC	CCTATGGCCCTCATTCTCAC	
10	*IL6ra*	NM_010559	AAGGAGGAGCTTGACCTTGG	CTCGTGGTTGGCAGAGTCTT	
11	*Tnfrsf1a (Tnfr1)*	NM_011609	CTTCATTCACGAGCGTTGTC	GGATGTATCCCCATCAGCAG	
12	*Tnfrsf1b (Tnfr2)*	NM_011610	TGAACTGCATCATCCTGGTG	GCACCTTGGCATCTCTTTGT	
13	*Casp9*	NM_015733	AGATCAGGGGACATGCAGAT	AGGAAGGGCAGAAGTTCACA	
14	*Tlr1*	NM_001276445	ATGCACAGCTCCTTGGTTTT	CTCTGCTCGCCTGAGTTCTT	
15	*Tlr2*	NM_011905	CATCACCGGTCAGAAAACAA	CCAAAGAGCTCGTAGCATCC	

16	*Tlr4*	NM_021297	ACACCAGGAAGCTTGAATCC	TGTCATCAGGGACTTTGCTG	
17	*Tlr6*	NM_011604	GGTACCGTCAGTGCTGGAAA	TCTGTCTTGGCTCATGTTGC	
18	*Cd14*	NM_009841	GCCTTTCTCGGAGCCTATCT	CAGAAGCAACAGCAACAAGC	
19	*Cd36*	NM_001159555	GCAAAGAACAGCAGCAAAATC	TCCTCGGGGTCCTGAGTTAT	
20	*Fatp1*	NM_011977	GGTGGTACTGCGCAAGAAGT	AGCGGCAGATTTCACCTATG	
21	*Adgre1 (F4/80)*	NM_010130	GGATGTACAGATGGGGGATG	CATAAGCTGGGCAAGTGGTA	
22	*Cd11b (Mac1)*	NM_001082960	AGCCCCACACTAGCATCAAG	TCCATGTCCACAGAGCAAAG	
23	*Itgax (Cd11c)*	NM_021334	CACTCAGTGACTGCCCAAAA	CCTCAAGACAGGACATCGCT	
24	*Cd206*	NM_008625	ACCTGGCAAGTATCCACAGC	AACCAATCCACAGCTCATCA	
25	*Cd4*	NM_013488	GCAGCATGGCAAAGGTGTAT	AAACGATCAAACTGCGAAGG	
26	*Cd8a*	NM_001081110	TCAGTTCTGTCGTGCCAGTC	GTGCACAGGTGAGGGAGTTC	
27	*Cd19*	NM_009844	TGTCTCCGAGGAAACCTGAC	CCATCCACCAGTTCTCAACA	
28	*Myh1*	NM_030679	GACCAAGGAGGAGGAACAGC	TCGTCTAGCTGGCGTGAGTA	
29	*Myh2*	NM_001039545	TCTTCTCTGGGGCACAAACT	CTTCTTGGCACCTTTCTTGG	
30	*Myh4*	NM_010855	CCAGACCGTGTCAGCTCTCT	GGGTGCTCTTCAAGTTGGTC	
31	*Myh7*	NM_080728	CTCCCAAGGAGAGACGACTG	AAGCCCAGCACGTCAAAG	
